# Associations Among Glycemic Variability, Hypomagnesemia, Thyroid Function, and Albuminuria in Patients With Type 2 Diabetes Mellitus: A Cross-Sectional Study

**DOI:** 10.7759/cureus.117117

**Published:** 2026-09-28

**Authors:** Shoaib Asghar, Kashif Shaikh, Ahmed Nisar, Sameera Aftab, Rabia Qadeer, Abdul Wahab

**Affiliations:** 1 General Internal Medicine, Sheikh Zayed Medical College/Hospital, Rahim Yar Khan, PAK; 2 General Internal Medicine, Luton and Dunstable University Hospital, Bedfordshire Hospitals NHS Foundation Trust, Luton, GBR

**Keywords:** continuous glucose monitoring (cgm), diabetic kidney disease (dkd), free thyroxine (ft4), free triiodothyronine (ft3), glucose time in range (tir), glycated hemoglobin (hba1c), mean amplitude of glycemic excursions (mage), thyroid-stimulating hormone (tsh), type 2 diabetes mellitus (t2dm), urinary albumin-to-creatinine ratio (uacr)

## Abstract

Background

Albuminuria is an important marker of diabetic kidney disease (DKD) and is associated with adverse renal and cardiovascular outcomes in people with type 2 diabetes mellitus (T2DM). Although sustained hyperglycemia is a recognized contributor to DKD, glycated hemoglobin (HbA1c) does not fully capture short-term glycemic variability. Emerging evidence suggests that hypomagnesemia and altered thyroid function may be associated with albuminuria, but their combined relationships with albuminuria and the possible statistical mediating role of thyroid hormones remain unclear. This study was conducted to identify the associations of albuminuria with glycemic variability, serum magnesium, and thyroid function in T2DM patients.

Materials and methods

A cross-sectional study included 330 adults aged 30-84 years with T2DM of more than five years' duration. Continuous glucose monitoring (CGM)-derived measures, including mean glucose, glucose standard deviation (SD), coefficient of variation (CV), time in range (TIR), and mean amplitude of glycemic excursions (MAGE), were assessed alongside HbA1c, serum magnesium, free triiodothyronine (FT3), free thyroxine (FT4), thyroid-stimulating hormone (TSH), and urinary albumin-to-creatinine ratio (UACR). Associations with albuminuria were examined using univariable and multivariable logistic regression, with additional correlation and exploratory regression-based mediation analysis. Ethical approval and written informed consent were obtained.

Results

Among 330 participants, 208 (63%) had normal UACR, 98 (29.7%) had microalbuminuria, and 24 (7.3%) had macroalbuminuria. Hypomagnesemia was associated with more than twofold higher odds of albuminuria in univariable analysis (odds ratio (OR)=2.26; 95% confidence interval (CI): 1.38-3.70; p=0.001). In Model IV, which was adjusted for demographic variables, higher HbA1c (OR=1.66; 95% CI: 1.34-2.05; p<0.001), SD (OR=1.63; 95% CI: 1.18-2.26; p=0.003), CV (OR=1.06; 95% CI: 1.01-1.10; p=0.016), and MAGE (OR=1.25; 95% CI: 1.07-1.45; p=0.005) were independently associated with higher odds of albuminuria. In contrast, higher FT3 was inversely associated with albuminuria (OR=0.46; 95% CI: 0.28-0.74; p=0.001). TIR, FT4, and TSH were not significantly associated with albuminuria in univariable analysis. HbA1c was negatively correlated with FT3 (ρ=-0.133; p=0.016). Similarly, SD was negatively correlated with FT3 (ρ=-0.227; p<0.001). Exploratory mediation analysis indicated that FT3 statistically accounted for 6.9% of the association between HbA1c and albuminuria and 21.4% of the association between glucose SD and albuminuria.

Conclusion

Greater glycemic variability and lower FT3 levels were independently associated with albuminuria in patients with T2DM, while hypomagnesemia was associated with albuminuria in univariable analysis. Exploratory mediation analysis suggested that FT3 statistically accounted for a modest proportion of the associations of HbA1c and glucose SD with albuminuria. These findings indicate that CGM-derived variability measures, alongside magnesium status and thyroid function, may provide complementary information in the assessment of DKD. Prospective multicenter studies are required to clarify the temporal relationships and clinical significance of these findings.

## Introduction

Diabetic kidney disease (DKD) or diabetic nephropathy (DN) is one of the most frequent microvascular complications of diabetes mellitus and has been reported in nearly 40% of patients with type 2 diabetes mellitus (T2DM). As a major cause of end-stage renal disease (ESRD), DKD represents a significant global burden in both developed and developing countries by reducing quality of life and life expectancy [1]. The complex mechanism of DKD remains underexplored, as several pathways and mediators are involved in its development. Albuminuria, a primary indicator of DKD, is also a significant risk factor for cardiovascular morbidity and mortality. Therefore, exploring the complex interaction between metabolic and hemodynamic factors is vital to reduce the prevalence and progression of DKD [1,2].

Optimal glycemic control is important in reducing microvascular risk and preventing the onset and progression of DKD, while chronic hyperglycemia leads to renal injury through multiple metabolic and inflammatory mechanisms [1-3]. The continuous glucose monitoring (CGM) system is a valuable emerging tool for assessing not only blood glucose variability but also collective glycemic status, furnishing a more comprehensive exploration of glucose control than traditional computations alone. Limited and conflicting literature related to the investigation of the relationship between DKD and CGM-derived glycemic indices is available so far. Some studies have reported a significant association of severity of albuminuria with glucose variability, while other researchers have found insufficient evidence to conclude such a relationship. These inconsistent findings emphasize the call for further investigations to accurately identify the function of glycemic variability in the development of DKD [4].

Abnormal serum magnesium levels are a common concern among patients with T2DM. The presence of microvascular or macrovascular complications, longer duration of diabetes, and poor glycemic control may increase the likelihood of hypomagnesemia [5]. Previous studies have also reported a significant association between hypomagnesemia, poor glycemic control, and diabetes-related complications. The overall prevalence percentage of hypomagnesemia in T2DM patients ranges from 13.5% to 47.7%, with a prevalence ratio of 32-34 per 100 T2DM patients residing in the South Asian region [5-7].

Thyroid hormones play an important role in regulating metabolic processes and maintaining the physiological functions of several organs. Recent literature have suggested an association between altered thyroid hormone levels and albuminuria in patients with T2DM [8]. Additionally, a significant association between glycemic status, thyroid function, and albuminuria has also been reported [4]. Although separate associations of glycemic variability, serum magnesium, thyroid hormones, and albuminuria have been described in the literature, studies examining these factors together remain limited, particularly in South Asian populations [8]. These findings also raised a hypothesis that thyroid hormones may mediate the associations of albuminuria with serum magnesium and glycemic levels.

Therefore, the primary objective of this study was to investigate the associations of albuminuria with CGM-derived glycemic metrics, serum magnesium, and thyroid hormones in patients with T2DM. Secondary analyses examined correlations of thyroid hormones with CGM-derived metrics and serum magnesium. Exploratory mediation analysis evaluated the statistical mediating role of thyroid hormones in the associations between CGM-derived metrics or serum magnesium and albuminuria.

By combining CGM-derived measures, thyroid hormone indices, serum magnesium levels, and albuminuria, the present study contributes to the existing literature on factors associated with DKD in T2DM.

## Materials and methods

Study design

A cross-sectional observational study was conducted among 330 adults aged 30-84 years with T2DM who attended the Department of Medicine and the outpatient diabetes clinic of Sheikh Zayed Hospital, Rahim Yar Khan, Pakistan, between July 2024 and July 2025. Ethical approval was obtained from the Institutional Review Board (IRB) of Sheikh Zayed Medical College/Hospital (approval number: 275/IRB/SZMC/SZH), and written informed consent was obtained from all participants. The research proforma used for data collection is shown in the Appendices.

Sample size

The sample size was estimated prospectively using an expected albuminuria prevalence of approximately 31% based on previous Pakistani studies [9], with a 95% confidence level and 5% absolute precision, giving a required sample of approximately 330 participants. A total of 657 consecutive patients were screened during the study period, of whom 330 fulfilled the inclusion and exclusion criteria and had the required study measurements available for analysis.

Selection criteria

The inclusion of patients in this study was based on the following criteria: (1) age ≥30 and ≤84 years, (2) confirmed T2DM for more than five years' duration, (3) availability of CGM-derived data, and (4) availability of thyroid function tests, serum magnesium, and urinary albumin-to-creatinine ratio (UACR) results. Patients with type 1 diabetes mellitus, with severe liver disease, with dialysis-dependent renal failure, with previous thyroid surgery or active thyroid disease treatment, and who were pregnant or lactating were excluded from this study.

There were no missing values among the 330 participants included in the final analysis; patients without the required study measurements were excluded during eligibility screening.

Covariates

Based on theoretical relevance, covariate selection of demographic variables included age, gender, body mass index (BMI), smoking status, systolic blood pressure (SBP), diastolic blood pressure (DBP), and hypertension history. Duration of diabetes was considered as a disease-related covariate. Hypomagnesemia was considered as a binary covariate and was defined as serum magnesium <1.70 mg/dL; values of 1.70-2.20 mg/dL were classified as the normal reference range. Laboratory investigations including glycated hemoglobin (HbA1c), serum magnesium, serum creatinine, blood urea nitrogen (BUN), estimated glomerular filtration rate (eGFR), cholesterol, triglycerides, high-density lipoprotein cholesterol (HDL-C), low-density lipoprotein cholesterol (LDL-C), uric acid, free triiodothyronine (FT3), free thyroxine (FT4), and thyroid-stimulating hormone (TSH) were recorded.

CGM data were obtained using the FreeStyle Libre/Libre 3 system by Abbott Diabetes Care (Witney, United Kingdom). The CGM-based metrics, including mean glucose, standard deviation (SD) glucose, coefficient of variation (CV) glucose, time in range (TIR), and mean amplitude of glycemic excursions (MAGE), were collected. Medication information including use of insulin, sulfonylureas, benzoic acid derivative, biguanide, dipeptidyl peptidase-4 (DPP-4) inhibitor, glycosidase inhibitor, thiazolidinedione (TZD), glucagon-like peptide-1 (GLP-1) receptor agonist, sodium-glucose cotransporter 2 (SGLT-2) inhibitor, β-receptor blocker, calcium channel blockers (CCBs), angiotensin-converting enzyme (ACE) inhibitor, angiotensin II receptor blockers (ARBs), mineralocorticoid receptor antagonist (MRA), diuretic, and α-receptor blocker was also gathered and included in the analysis (research proforma attached in the Appendices). 

Dependent variable

Albuminuria was classified according to UACR as follows: normal albuminuria as <30 mg/g, microalbuminuria as 30-299 mg/g, and macroalbuminuria as ≥300 mg/g. For UACR assessment, patients provided a clean-catch, mid-stream urine sample from the first morning void, following the routine collection protocol used at the study center. UACR was used to classify cross-sectional albuminuria status at the study assessment; the study was not designed to establish longitudinal persistence of albuminuria.

For univariate and multivariate binary logistic regression analyses examining factors associated with albuminuria, participants were categorized into two classes: Class I with albuminuria, defined as microalbuminuria or macroalbuminuria with a UACR ≥30 mg/g, and Class II featuring normal albumin excretion, defined as a UACR <30 mg/g. For analysis examining factors associated with macroalbuminuria, participants were categorized as having macroalbuminuria, defined as a UACR ≥300 mg/g, or having microalbuminuria or normal albumin excretion, defined as a UACR <300 mg/g.

Statistical methods

IBM SPSS Statistics for Windows, Version 23.0 (IBM Corp., Armonk, New York, United States), was used for the analysis of study data. The Shapiro-Wilk test was used to assess the normality of continuous variables. Comparison analysis among three classes of albuminuria was performed by using the chi-squared test for categorical variables, the Kruskal-Wallis H rank-sum test for non-normal continuous covariates, and one-way analysis of variance (ANOVA) for normally distributed continuous covariates.

Number with percentage (n (%)), mean±standard deviation (SD), and median with interquartile range (IQR) were calculated for categorical variables, normally distributed variables, and non-normally distributed variables, respectively. Bivariate correlation analysis of thyroid hormone indices with CGM-based metrics and magnesium level was conducted through Spearman's rank correlation test.

To examine and identify the significant risk factors of albuminuria and macroalbuminuria, a univariate binary logistic regression modeling technique was executed. The explanatory variables included were demographic characteristics, disease- and medication-related variables, biochemical factors, CGM-based metrics, serum magnesium, and thyroid hormones.

Variables from serum magnesium, thyroid function, or CGM-based metrics that were significant in univariable analyses were then evaluated in four separate domain-specific multivariable logistic regression models: Model I adjusted for biochemical variables, Model II for medication-related variables, Model III for disease-related variables, and Model IV for demographic variables. These models were used as separate adjustment domains rather than as sequential cumulative models. Each significant CGM-derived metric, FT3, and serum magnesium was evaluated separately within each adjustment model rather than being entered simultaneously into a single exposure model.

Regression-based mediation analysis was performed to examine the potential mediation produced by thyroid hormones in the association of albuminuria with serum magnesium and glycemic level. Albuminuria was considered as a binary outcome variable, HbA1c, mean, SD, and CV of glucose, MAGE, and serum magnesium were taken as exposure variables, and FT3, FT4, and TSH were regarded as mediators. A simple mediation model with a 95% confidence interval (CI) and without adjustment of any covariate was executed. A probability value (p-value) of <0.05 is considered statistically significant. Given the cross-sectional design, the mediation analysis was treated as exploratory statistical mediation and was not interpreted as establishing temporal or causal pathways.

## Results

Basic descriptive statistics, including mean, median, and SD, were calculated. The findings showed the significance of explanatory variables based on the chi-squared test, Kruskal-Wallis H rank-sum test, and ANOVA. Univariate and multivariate binary logistic regression models identified the significant explanatory variables. Mediation analysis examined indirect statistical associations through thyroid hormones in the association of albuminuria with serum magnesium and glycemic measures.

Descriptive characteristics

Based on UACR, the total sample of 330 T2DM patients was classified into a macroalbuminuria class (n=24), a microalbuminuria class (n=98), and a normal albuminuria class (n=208). The distribution of basic descriptive characteristics for the three classes of T2DM patients is shown in Table 1. 

**Table 1:** Clinical descriptive statistics F: one-way ANOVA test statistic; χ²: chi-squared test statistic; H: Kruskal-Wallis H test statistic T2DM: type 2 diabetes mellitus; SD: standard deviation; BMI: body mass index; SBP: systolic blood pressure; DBP: diastolic blood pressure; HDL-C: high-density lipoprotein cholesterol; LDL-C: low-density lipoprotein cholesterol; BUN: blood urea nitrogen; eGFR: estimated glomerular filtration rate; UACR: urinary albumin-to-creatinine ratio; DPP-4: dipeptidyl peptidase-4; TZD: thiazolidinedione; GLP-1: glucagon-like peptide-1; SGLT-2: sodium-glucose cotransporter 2; CCB: calcium channel blocker; ACE: angiotensin-converting enzyme; ARB: angiotensin II receptor blocker; MRA: mineralocorticoid receptor antagonist; IQR: interquartile range; ANOVA: analysis of variance

Variables	Normal albuminuria (n=208)	Microalbuminuria (n=98)	Macroalbuminuria (n=24)	Total T2DM patients (N=330)	Test statistic	P-value
Age (years), mean±SD	57.9±10.9	58.3±9.5	59.1±11.2	58.1±10.5	F=0.16	0.850
Sex, n (%)	-	-	-	-	χ²=0.53	0.769
Female sex	99 (47.6)	45 (45.9)	13 (54.2)	157 (47.6)	χ²=72.20	<0.001
Male sex	109 (52.4)	53 (54.1)	11 (45.8)	173 (52.4)	χ²=83.83	<0.001
BMI (kg/m^2^), mean±SD	26.1±3.5	25.3±3.3	25.9±2.9	25.8±3.4	F=2.07	0.127
SBP (mmHg), mean±SD	132.4±13.5	132.6±13.2	127.3±10.9	132.1±13.3	F=1.69	0.185
DBP (mmHg), mean±SD	78.8±8.9	79.9±9.1	84.1±10.2	79.4±9.1	F=3.85	0.022
Smoking, n (%)	-	-	-	-	χ²=7.55	0.110
Smoking: never	126 (60.6)	59 (60.2)	13 (54.2)	198 (60)	χ²=97.85	<0.001
Smoking: ex-smoker	41 (19.7)	15 (15.3)	9 (37.5)	65 (19.7)	χ²=26.71	<0.001
Smoking: current smoker	41 (19.7)	24 (24.5)	2 (8.3)	67 (20.3)	χ²=34.24	<0.001
History of hypertension, n (%)	-	-	-	-	χ²=6.19	0.045
Hypertension: no	113 (54.3)	39 (39.8)	10 (41.7)	162 (49.1)	χ²=104.48	<0.001
Hypertension: yes	95 (45.7)	59 (60.2)	14 (58.3)	168 (50.9)	χ²=58.82	<0.001
Duration of T2DM (years), median (IQR)	8.3 (5.6-11.8)	9.05 (4.2-11.8)	8.0 (5.2-9.2)	8.4 (5.3-11.7)	H=2.45	0.294
Cholesterol (mmol/L), mean±SD	4.71±0.82	4.68±0.84	4.80±0.91	4.71±0.83	F=0.21	0.811
HDL-C (mmol/L), mean±SD	1.03±0.21	1.02±0.21	0.90±0.16	1.02±0.21	F=4.32	0.014
LDL-C (mmol/L), mean±SD	2.44±0.65	2.67±0.68	2.41±0.84	2.51±0.68	F=4.26	0.015
Triglycerides (mmol/L), mean±SD	1.63±0.59	1.78±0.63	1.93±0.61	1.70±0.61	F=4.01	0.019
Uric acid (μmol/L), mean±SD	305.68±91.32	323.68±77.72	356.99±91.00	314.76±88.39	F=4.43	0.013
BUN (mmol/L), mean±SD	5.82±1.46	6.17±1.67	6.66±1.70	5.98±1.56	F=4.23	0.015
Creatinine (μmol/L), median (IQR)	76.7 (65.9-93.7)	89.8 (75.8-104.3)	102.2 (70.4-113.6)	84.0 (67.5-99.4)	H=5.53	0.063
eGFR (mL/min/1.73 m^2^), median (IQR)	92.8 (77.9-100.6)	85.5 (72.1-94.6)	82.9 (60.1-111.1)	89.9 (75.0-99.7)	H=0.24	0.887
UACR (mg/g), median (IQR)	15.4 (10.2-21.7)	144.9 (71.1-214.3)	581.85 (418.2-846.5)	22.20 (13.0-113.8)	H=57.37	<0.001
Hypomagnesemia (mg/dl) (yes), n (%)	44 (21.2)	40 (40.8)	6 (25)	90 (27.3)	χ²=13.05	0.001
Insulin use, n (%)	112 (53.8)	50 (51)	16 (66.7)	178 (53.9)	χ²=1.90	0.386
Sulfonylureas use, n (%)	33 (15.9)	18 (18.4)	6 (25)	57 (17.3)	χ²=1.37	0.503
Benzoic acid derivative use, n (%)	23 (11.1)	12 (12.2)	5 (20.8)	40 (12.1)	χ²=1.93	0.381
Biguanide use, n (%)	135 (64.9)	65 (66.3)	14 (58.3)	214 (64.8)	χ²=0.54	0.763
DPP-4 inhibitor use, n (%)	50 (24)	28 (28.6)	6 (25)	84 (25.5)	χ²=0.72	0.696
Glycosidase inhibitor use, n (%)	72 (34.6)	33 (33.7)	10 (41.7)	115 (34.8)	χ²=0.56	0.757
TZD use, n (%)	16 (7.7)	11 (11.2)	5 (20.8)	32 (9.7)	χ²=4.62	0.100
GLP-1 receptor agonist use, n (%)	16 (7.7)	8 (8.2)	5 (20.8)	29 (8.8)	χ²=4.70	0.095
SGLT-2 inhibitor use, n (%)	19 (9.1)	6 (6.1)	5 (20.8)	30 (9.1)	χ²=5.05	0.080
β-Receptor blocker use, n (%)	25 (12)	9 (9.2)	6 (25)	40 (12.1)	χ²=4.53	0.104
CCB use, n (%)	50 (24)	22 (22.4)	7 (29.2)	79 (23.9)	χ²=0.481	0.786
ACE inhibitor use, n (%)	20 (9.6)	11 (11.2)	5 (20.8)	36 (10.9)	χ²=2.80	0.247
ARB use, n (%)	51 (24.4)	31 (31.6)	7 (29.2)	89 (27)	χ²=1.78	0.412
MRA use, n (%)	22 (10.6)	14 (14.3)	6 (25)	42 (12.7)	χ²=4.34	0.114
Diuretic use, n (%)	19 (9.1)	10 (10.2)	6 (25)	35 (10.6)	χ²=5.74	0.057
α-Receptor blocker use, n (%)	21 (10.1)	11 (11.2)	5 (20.8)	37 (11.2)	χ²=2.49	0.288

The mean±SD age of patients with T2DM was 58.13±10.53 including 173 (52.4%) males and 157 (47.6%) females. Statistical tests of comparison showed that history of hypertension, DBP, HDL-C, LDL-C, triglycerides, uric acid, BUN, UACR, and hypomagnesemia (p≤0.05) were significantly different among the three classes. Other variables, including age, sex, smoking status, BMI, SBP, duration of T2DM, cholesterol, creatinine, eGFR, and use of insulin, sulfonylureas, benzoic acid derivative, biguanide, DPP-4 inhibitor, glycosidase inhibitor, TZD, GLP-1 receptor agonist, SGLT-2 inhibitor, β-receptor blocker, CCB, ACE, ARB, MRA, diuretic, and α-receptor blocker, were not significantly different (p>0.05) among the three classes.

Comparative analysis of CGM-based metrics, magnesium level, and thyroid function

Table 2 presented the descriptive statistics of CGM-based metrics, magnesium level, and thyroid function for the whole sample of T2DM patients and each class of albuminuria separately. The analysis showed the deviation of HbA1c (p=0.021), mean of glucose (p=0.001), SD of glucose (p=0.018), CV of glucose (p=0.002), MAGE (p=0.015), FT3 (p=0.007), and magnesium level (p=0.025) among macroalbuminuria, microalbuminuria, and normal albuminuria classes. The findings further found that the distribution of TIR, FT4, and TSH was similar (p>0.05) among all three albuminuria classes. 

**Table 2:** Distribution of CGM-based metrics, magnesium level, and thyroid function F: one-way ANOVA test statistic; χ²: chi-squared test statistic; H: Kruskal-Wallis H test statistic CGM: continuous glucose monitoring; T2DM: type 2 diabetes mellitus; HbA1c: glycated hemoglobin; SD: standard deviation; IQR: interquartile range; TIR: time in range; CV: coefficient of variation; MAGE: mean amplitude of glycemic excursions; FT3: free triiodothyronine; FT4: free thyroxine; TSH: thyroid-stimulating hormone; ANOVA: analysis of variance

Variables	Normal (n=208)	Microalbuminuria (n=98)	Macroalbuminuria (n=24)	Total T2DM patients (N=330)	Test statistic	P-value
HbA1c (%), median (IQR)	8.7 (7.9-9.6)	9.7 (8.9-10.3)	8.9 (8.5-9.5)	9.0 (8.1-9.9)	H=7.73	0.021
Mean of glucose (mmol/L), mean±SD	9.2±1.8	9.9±1.7	9.0±1.5	9.4±1.8	F=7.14	0.001
SD of glucose (mmol/L), median (IQR)	2.1 (1.5-2.6)	2.4 (1.8-2.8)	2.5 (1.3-2.8)	2.2 (1.6-2.7)	H=8.03	0.018
TIR (%), median (IQR)	60.5 (49.2-75.5)	59.8 (49.0-72.4)	57.1 (50.2-68.4)	59.9 (49.3-75.1)	H=1.56	0.459
CV of glucose (%), mean±SD	22.3±5.7	24.5±4.7	21.5±5.0	22.9±5.4	F=6.25	0.002
MAGE (mmol/L), mean±SD	5.0±1.6	5.5±1.4	5.6±1.1	5.2±1.5	F=4.28	0.015
FT3 (pmol/L), median (IQR)	4.3 (3.9-4.6)	4.1 (3.7-4.5)	4.0 (3.8-4.3)	4.2 (3.9-4.5)	H=9.92	0.007
FT4 (pmol/L), mean±SD	15.9±2.1	15.7±1.9	15.5±2.6	15.8±2.1	F=0.33	0.720
TSH (mIU/L), mean±SD	2.1±0.8	2.0±0.8	1.8±0.9	2.1±0.8	F=1.53	0.219
Serum magnesium (mg/dL), mean±SD	1.9±0.2	1.8±0.2	1.9±0.2	1.8±0.2	F=3.73	0.025

Bivariate correlation analysis between CGM-based metrics, magnesium level, and thyroid hormones

Spearman's rank correlation measure (ρ) of thyroid hormone indices with CGM-based metrics and magnesium level is presented in Table 3. The analysis showed that HbA1c (p=0.016) and SD of glucose (p<0.001) were negatively correlated with FT3. Moreover, MAGE (p=0.038) showed a significant negative correlation with TSH. All other CGM-based metrics and serum magnesium were uncorrelated with FT3 for the observed data. Apart from the significant inverse correlation between MAGE and TSH, no other significant correlations involving FT4 or TSH were observed.

**Table 3:** Correlation between CGM-based metrics, magnesium level, and thyroid hormone level HbA1c: glycated hemoglobin; SD: standard deviation; TIR: time in range; CV: coefficient of variation; MAGE: mean amplitude of glycemic excursions; FT3: free triiodothyronine; FT4: free thyroxine; TSH: thyroid-stimulating hormone; rho (ρ): Spearman's rank correlation coefficient

Variables	FT3 (pmol/L)		FT4 (pmol/L)		TSH (mIU/L)	
	ρ	P-value	ρ	P-value	ρ	P-value
HbA1c (%)	-0.133	0.016	-0.072	0.192	-0.076	0.171
Mean of glucose (mmol/L)	0.012	0.832	-0.043	0.434	-0.048	0.383
SD of glucose (mmol/L)	-0.227	<0.001	-0.043	0.440	0.041	0.459
TIR (%)	0.074	0.178	-0.001	0.990	-0.017	0.763
CV of glucose (%)	-0.022	0.684	-0.068	0.220	0.077	0.161
MAGE (mmol/L)	0.009	0.867	-0.075	0.173	-0.114	0.038
Serum magnesium (mg/dL)	-0.012	0.826	0.003	0.956	-0.011	0.839

Identification of factors associated with albuminuria and macroalbuminuria using univariate binary logistic regression

The univariate binary logistic regression analysis presented in Table 4 was performed to examine the effect of demographic, disease-related, biochemical, and medication information variables on albuminuria and macroalbuminuria. The investigation was further extended to identify the significant associations among CGM-based metrics, magnesium level, and thyroid hormones. The results revealed a significant association of history of hypertension (p=0.013), LDL-C (p=0.021), triglycerides (p=0.011), uric acid (p=0.016), BUN (p=0.012), creatinine (p<0.001), eGFR (p=0.042), and hypomagnesemia (p=0.001) with the risk of albuminuria. The present study found no significant association of albuminuria with any medication-related covariate. Among CGM-based metrics, mean of glucose (p=0.004), SD of glucose (p=0.006), CV of glucose (p=0.013), and MAGE (p=0.004) were associated with the odds of albuminuria. Additionally, HbA1c (p<0.001) was positively while FT3 (p=0.002) and serum magnesium (p=0.033) were inversely associated with the odds of albuminuria.

**Table 4:** The univariate logistic regression analysis to examine the association of albuminuria with CGM-based metrics, thyroid hormone indices, and magnesium level Statistical test: univariate binary logistic regression. Odds ratios are reported with 95% confidence intervals. OR: odds ratio; CI: confidence interval; BMI: body mass index; SBP: systolic blood pressure; DBP: diastolic blood pressure; T2DM: type 2 diabetes mellitus; HDL-C: high-density lipoprotein cholesterol; LDL-C: low-density lipoprotein cholesterol; BUN: blood urea nitrogen; eGFR: estimated glomerular filtration rate; DPP-4: dipeptidyl peptidase-4; TZD: thiazolidinedione; GLP-1: glucagon-like peptide-1; SGLT-2: sodium-glucose cotransporter 2; CCB: calcium channel blocker; ACE: angiotensin-converting enzyme; ARB: angiotensin II receptor blocker; MRA: mineralocorticoid receptor antagonist; HbA1c: glycated hemoglobin; TIR: time in range; CV: coefficient of variation; MAGE: mean amplitude of glycemic excursions; FT3: free triiodothyronine; FT4: free thyroxine; TSH: thyroid-stimulating hormone

Variables	Odds of albuminuria		Odds of macroalbuminuria	
	OR (95% CI)	P-value	OR (95% CI)	P-value
Age (years)	1.01 (0.98-1.03)	0.628	1.01 (0.97-1.05)	0.65
Gender (male)	1.00 (0.64-1.57)	0.992	1.33 (0.58-3.06)	0.503
BMI (kg/m^2^)	0.94 (0.88-1.00)	0.062	1.00 (0.89-1.13)	0.97
SBP (mmHg)	0.99 (0.98-1.01)	0.576	0.97 (0.94-1.00)	0.068
DBP (mmHg)	1.02 (0.99-1.05)	0.097	1.06 (1.02-1.11)	0.009
Smoking (yes)	1.05 (0.79-1.39)	0.730	0.90 (0.53-1.54)	0.698
History of hypertension (yes)	1.77 (1.13-2.79)	0.013	1.38 (0.59-3.21)	0.452
Duration of T2DM (years)	0.98 (0.94-1.03)	0.527	0.94 (0.85-1.03)	0.202
Cholesterol (mmol/L)	0.99 (0.75-1.29)	0.911	1.15 (0.70-1.90)	0.579
HDL-C (mmol/L)	0.48 (0.16-1.41)	0.182	0.04 (0.01-0.39)	0.004
LDL-C (mmol/L)	1.49 (1.06-2.09)	0.021	0.79 (0.43-1.46)	0.454
Triglycerides (mmol/L)	1.63 (1.12-2.37)	0.011	1.95 (1.00-3.80)	0.049
Uric acid (μmol/L)	1.01 (1.00-1.02)	0.016	1.01 (1.00-1.02)	0.016
BUN (mmol/L)	1.21 (1.04-1.39)	0.012	1.35 (1.03-1.77)	0.029
Creatinine (μmol/L)	1.02 (1.01-1.03)	<0.001	1.03 (1.01-1.05)	0.004
eGFR (mL/min/1.73 m^2^)	0.99 (0.98-1.00)	0.042	0.99 (0.97-1.02)	0.620
Hypomagnesemia (yes)	2.26 (1.38-3.70)	0.001	0.88 (0.34-2.29)	0.795
Insulin use (yes)	1.01 (0.65-1.58)	0.965	1.78 (0.74-4.28)	0.199
Sulfonylurea use (yes)	1.29 (0.73-2.32)	0.378	1.67 (0.63-4.40)	0.303
Benzoic acid derivative use (yes)	1.30 (0.67-2.55)	0.440	2.04 (0.72-5.80)	0.182
Biguanide use (yes)	0.99 (0.62-1.59)	0.978	0.74 (0.32-1.73)	0.489
DPP-4 inhibitor use (yes)	1.22 (0.74-2.03)	0.441	0.97 (0.37-2.54)	0.958
Glycosidase inhibitor use(yes)	1.03 (0.64-1.64)	0.908	1.37 (0.59-3.18)	0.468
TZD use (yes)	1.81 (0.87-3.77)	0.112	2.72 (0.94-7.86)	0.065
GLP-1 receptor agonist use (yes)	1.43 (0.66-3.09)	0.361	3.09 (1.06-9.01)	0.039
SGLT-2 inhibitor use (yes)	0.99 (0.45-2.15)	0.971	2.96 (1.02-8.59)	0.046
β-Receptor blocker use (yes)	1.03 (0.52-2.03)	0.941	2.67 (0.99-7.18)	0.052
CCB use (yes)	0.99 (0.58-1.67)	0.956	1.34 (0.53-3.36)	0.534
ACE inhibitor use (yes)	1.42 (0.71-2.86)	0.327	2.33 (0.82-6.69)	0.114
ARB use (yes)	1.39 (0.85-2.29)	0.191	1.13 (0.45-2.81)	0.801
MRA use (yes)	1.66 (0.86-3.18)	0.129	2.50 (0.93-6.71)	0.069
Diuretic use (yes)	1.50 (0.74-3.04)	0.259	3.18 (1.17-8.66)	0.023
α-Receptor blocker use (yes)	1.34 (0.67-2.69)	0.403	2.25 (0.79-6.45)	0.130
HbA1c (%)	1.70 (1.38-2.09)	<0.001	1.06 (0.76-1.47)	0.752
Mean of glucose (mmol/L)	1.21 (1.06-1.37)	0.004	0.88 (0.69-1.11)	0.272
SD of glucose (mmol/L)	1.55 (1.14-2.11)	0.006	1.17 (0.69-2.04)	0.588
TIR (%)	0.99 (0.98-1.01)	0.786	0.99 (0.97-1.01)	0.388
CV of glucose (%)	1.06 (1.01-1.11)	0.013	0.95 (0.88-1.03)	0.179
MAGE (mmol/L)	1.25 (1.03-1.46)	0.004	1.18 (0.89-1.55)	0.179
FT3 (pmol/L)	0.49 (0.31-0.77)	0.002	0.66 (0.29-1.49)	0.138
FT4 (pmol/L)	0.96 (0.86-1.07)	0.476	0.94 (0.77-1.14)	0.531
TSH (mIU/L)	0.85 (0.65-1.11)	0.224	0.66 (0.40-1.10)	0.109
Serum magnesium (mg/dL)	0.29 (0.09-0.90)	0.033	2.28 (0.27-19.17)	0.449

Concerning macroalbuminuria, the study findings showed that DBP (p=0.009) was a highly significant factor among demographic characteristics. Based on biochemical characteristics, HDL-C (p=0.004) showed an inverse association with the odds of macroalbuminuria, whereas triglycerides (p=0.049), uric acid (p=0.016), BUN (p=0.029), and creatinine (p=0.004) showed positive associations with the odds of macroalbuminuria. Medical treatment, including GLP-1 receptor agonist (p=0.039), SGLT-2 inhibitor (p=0.046), and diuretic (p=0.023), was also significantly associated with the odds of macroalbuminuria.

Multivariate logistic regression adjusted models

Multivariable logistic regression analysis examined four separate adjustment models. Model I adjusted for biochemical variables, Model II for medication-related variables, Model III for duration of T2DM, and Model IV for demographic variables. Across these models, HbA1c, mean glucose, glucose SD, CV, and MAGE remained associated with higher odds of albuminuria, while FT3 remained inversely associated with albuminuria. Serum magnesium was inversely associated with albuminuria in Models I-III, but the association was attenuated and was no longer statistically significant in Model IV (p=0.060). No significant associations with macroalbuminuria were observed for these variables in the adjusted models (Table 5).

**Table 5:** Association of albuminuria and macroalbuminuria with significant CGM-based metrics, thyroid hormone indices, and magnesium level through multivariate logistic regression model Statistical test: multivariable binary logistic regression. Odds ratios are reported with 95% confidence intervals. Model I is adjusted for eight biochemical variables including serum creatinine, BUN, eGFR, cholesterol, triglycerides, HDL-C, LDL-C, and uric acid. Model II is adjusted for 16 medication information variables including use of insulin, sulfonylureas, benzoic acid derivative, biguanide, DPP-4 inhibitor, glycosidase inhibitor, TZD, GLP-1 receptor agonist, SGLT-2 inhibitor, β-receptor blocker, CCBs, ACE inhibitor, ARBs, MRA, diuretic, and α-receptor blocker. Model III is adjusted for one disease-related variable including duration of T2DM. Model IV is adjusted for seven demographic variables including age, gender, BMI, smoking status, SBP, DBP, and hypertension history. HbA1c: glycated hemoglobin; SD: standard deviation; CV: coefficient of variation; MAGE: mean amplitude of glycemic excursions; FT3: free triiodothyronine; BUN: blood urea nitrogen; eGFR: estimated glomerular filtration rate; HDL-C: high-density lipoprotein cholesterol; LDL-C: low-density lipoprotein cholesterol; PP-4: dipeptidyl peptidase-4; TZD: thiazolidinedione; GLP-1: glucagon-like peptide-1; SGLT-2: sodium-glucose cotransporter 2; CCB: calcium channel blocker; ACE: angiotensin-converting enzyme; ARB: angiotensin II receptor blocker; MRA: mineralocorticoid receptor antagonist; BMI: body mass index; SBP: systolic blood pressure; DBP: diastolic blood pressure; T2DM: type 2 diabetes mellitus

Variables	Odds of albuminuria		Odds of macroalbuminuria	
	OR (95% CI)	P-value	OR (95% CI)	P-value
HbA1c (per %)				
Model I	1.62 (1.31-2.01)	<0.001	0.91 (0.62-1.33)	0.622
Model II	1.68 (1.36-2.07)	<0.001	1.04 (0.71-1.50)	0.859
Model III	1.69 (1.38-2.09)	<0.001	1.04 (0.74-1.45)	0.834
Model IV	1.66 (1.34-2.05)	<0.001	0.97 (0.68-1.38)	0.857
Mean of glucose (mmol/L)				
Model I	1.16 (1.01-1.33)	0.039	0.79 (0.61-1.05)	0.101
Model II	1.23 (1.07-1.40)	0.003	0.86 (0.67-1.12)	0.271
Model III	1.21 (1.06-1.37)	0.005	0.87 (0.69-1.09)	0.240
Model IV	1.19 (1.04-1.36)	0.010	0.88 (0.69-1.12)	0.281
SD of glucose (mmol/L)				
Model I	1.47 (1.06-2.04)	0.021	1.21 (0.66-2.23)	0.541
Model II	1.58 (1.15-2.18)	0.005	1.22 (0.66-2.25)	0.532
Model III	1.56 (1.14-2.12)	0.005	1.19 (0.68-2.08)	0.546
Model IV	1.63 (1.18-2.26)	0.003	1.22 (0.67-2.22)	0.516
CV of glucose (%)				
Model I	1.06 (1.01-1.11)	0.008	0.95 (0.87-1.04)	0.276
Model II	1.07 (1.02-1.12)	0.005	0.96 (0.88-1.04)	0.330
Model III	1.06 (1.01-1.10)	0.012	0.95 (0.88-1.03)	0.199
Model IV	1.06 (1.01-1.10)	0.016	0.92 (0.84-1.00)	0.055
MAGE (mmol/L)				
Model I	1.24 (1.06-1.46)	0.009	1.09 (0.79-1.49)	0.600
Model II	1.27 (1.08-1.48)	0.004	1.26 (0.94-1.70)	0.123
Model III	1.26 (1.08-1.46)	0.004	1.19 (0.90-1.56)	0.219
Model IV	1.25 (1.07-1.45)	0.005	1.17 (0.88-1.54)	0.282
FT3 (pmol/L)				
Model I	0.56 (0.34-0.91)	0.019	0.76 (0.31-1.85)	0.541
Model II	0.46 (0.29-0.74)	0.001	0.57 (0.22-1.43)	0.227
Model III	0.48 (0.31-0.76)	0.002	0.65 (0.29-1.46)	0.294
Model IV	0.46 (0.28-0.74)	0.001	0.58 (0.23-1.44)	0.239
Serum magnesium (mg/dL)				
Model I	0.27 (0.08-0.92)	0.037	3.71 (0.36-38.06)	0.270
Model II	0.21 (0.06-0.72)	0.013	1.69 (0.15-19.44)	0.676
Model III	0.29 (0.09-0.91)	0.034	2.38 (0.28-19.91)	0.425
Model IV	0.32 (0.09-1.05)	0.060	2.47 (0.28-21.63)	0.413

Exploratory mediation analysis

Exploratory regression-based mediation analysis showed statistically significant indirect associations through FT3 for HbA1c and glucose SD, accounting for 6.9% and 21.4% of their respective associations with albuminuria. No statistically significant indirect associations through FT3 were observed for mean glucose, CV, MAGE, or serum magnesium, and no significant indirect associations through FT4 or TSH were identified. These findings are interpreted as exploratory statistical mediation rather than evidence of a temporal or causal pathway (Table 6).

**Table 6:** Mediating effects of thyroid hormones Statistical method: regression-based mediation analysis CI: confidence interval; HbA1c: glycated hemoglobin; SD: standard deviation; CV: coefficient of variation; MAGE: mean amplitude of glycemic excursions; FT3: free triiodothyronine; FT4: free thyroxine; TSH: thyroid-stimulating hormone

Exposure variable	Mediator	Effect	Estimate	95% CI	P-value	Proportion mediated (%)
HbA1c	FT3	Average indirect	0.000219	0.00001 to 0.0014	0.018	6.9
HbA1c	FT3	Average direct	0.0029	0.0005 to 0.0093	<0.001	6.9
HbA1c	FT3	Total	0.0032	0.0006 to 0.0106	<0.001	6.9
Mean of glucose	FT3	Average indirect	-0.0006	-0.0044 to 0.0014	0.578	-3.57
Mean of glucose	FT3	Average direct	0.0176	0.0092 to 0.02159	0.0012	-3.57
Mean of glucose	FT3	Total	0.0170	0.0089 to 0.0196	0.0024	-3.57
SD of glucose	FT3	Average indirect	0.0159	0.0033 to 0.0346	0.0112	21.4
SD of glucose	FT3	Average direct	0.0585	0.0047 to 0.087	0.0384	21.4
SD of glucose	FT3	Total	0.0745	0.0263 to 0.0981	0.0080	21.4
CV of glucose	FT3	Average indirect	0.0002	-0.0009 to 0.0011	0.6836	2.74
CV of glucose	FT3	Average direct	0.0067	0.0027 to 0.0078	0.0100	2.74
CV of glucose	FT3	Total	0.0069	0.0028 to 0.0078	0.0100	2.74
MAGE	FT3	Average indirect	0.00004	-0.0039 to 0.0038	0.9904	0.156
MAGE	FT3	Average direct	0.0315	0.0159 to 0.0376	0.0024	0.156
MAGE	FT3	Total	0.0315	0.0159 to 0.0378	0.0032	0.156
Serum magnesium	FT3	Average indirect	0.0090	-0.0288 to 0.0538	0.6412	-3.94
Serum magnesium	FT3	Average direct	-0.2384	-0.2715 to -0.0359	0.0280	-3.94
Serum magnesium	FT3	Total	-0.2294	-0.2527450 to -0.01742	0.0356	-3.94
HbA1c	FT4	Average indirect	0.00001	-0.0001 to 0.0002	0.7652	0.40
HbA1c	FT4	Average direct	0.0033	0.0006 to 0.0106	<0.001	0.40
HbA1c	FT4	Total	0.0033	0.0006 to 0.0106	<0.001	0.40
Mean of glucose	FT4	Average indirect	0.0001	-0.0009 to 0.0013	0.8112	0.86
Mean of glucose	FT4	Average direct	0.0164	0.0085 to 0.0194	0.0020	0.86
Mean of glucose	FT4	Total	0.0166	0.0089 to 0.0194	0.0016	0.86
SD of glucose	FT4	Average indirect	0.0006	-0.0030 to 0.0053	0.7804	0.78
SD of glucose	FT4	Average direct	0.0741	0.0260 to 0.0972	0.0076	0.78
SD of glucose	FT4	Total	0.0746	0.0266 to 0.0978	0.0080	0.78
CV of glucose	FT4	Average indirect	0.00008	-0.0003 to 0.0006	0.7164	1.20
CV of glucose	FT4	Average direct	0.0067	0.0026 to 0.0077	0.0104	1.20
CV of glucose	FT4	Total	0.0068	0.0028 to 0.0078	0.0100	1.20
MAGE	FT4	Average indirect	0.0004	-0.0018 to 0.003	0.6788	1.42
MAGE	FT4	Average direct	0.0308	0.0148 to 0.0366	0.0032	1.42
MAGE	FT4	Total	0.0312	0.0158 to 0.0367	0.0028	1.42
Serum magnesium	FT4	Average indirect	-0.0004	-0.0142 to 0.0145	0.9780	0.18
Serum magnesium	FT4	Average direct	-0.2275	-0.2515 to -0.0194	0.0352	0.18
Serum magnesium	FT4	Total	-0.2279	-0.2512 to -0.0177	0.0356	0.18
HbA1c	TSH	Average indirect	0.00004	-0.0001 to 0.0004	0.488	1.38
HbA1c	TSH	Average direct	0.0032	0.0006 to 0.0104	<0.001	1.38
HbA1c	TSH	Total	0.0032	0.0006 to 0.0106	<0.001	1.38
Mean of glucose	TSH	Average indirect	0.0003	-0.0005 to 0.0020	0.5684	1.58
Mean of glucose	TSH	Average direct	0.0163	0.0083 to 0.0192	0.0024	1.58
Mean of glucose	TSH	Total	0.0166	0.0085 to 0.0194	0.0016	1.58
SD of glucose	TSH	Average indirect	-0.0015	-0.0079 to 0.0026	0.5484	-1.96
SD of glucose	TSH	Average direct	0.0761	0.0282 to 0.0997	0.0068	-1.96
SD of glucose	TSH	Total	0.0746	0.0266 to 0.0978	0.0080	-1.96
CV of glucose	TSH	Average indirect	-0.0003	-0.0014 to 0.0002	0.2604	-5.03
CV of glucose	TSH	Average direct	0.0072	0.0032 to 0.0083	0.0072	-5.03
CV of glucose	TSH	Total	0.0068	0.0028 to 0.0078	0.0100	-5.03
MAGE	TSH	Average indirect	0.0012	-0.0016 to 0.005	0.4176	3.78
MAGE	TSH	Average direct	0.0300	0.0134 to 0.0359	0.0032	3.78
MAGE	TSH	Total	0.0312	0.0158 to 0.0367	0.0024	3.78
Serum magnesium	TSH	Average indirect	0.0031	-0.0172 to 0.0220	0.7532	-1.35
Serum magnesium	TSH	Average direct	-0.2310	-0.2579 to -0.0200	0.0376	-1.35
Serum magnesium	TSH	Total	-0.2279	-0.2510 to -0.0176	0.0356	-1.35

The direct effect analysis showed that HbA1c, mean, SD, and CV of glucose, MAGE, and serum magnesium were associated with albuminuria, independent of FT3, FT4, and TSH.

## Discussion

This cross-sectional study investigated the individual and collective relationships between glycemic variability, hypomagnesemia, thyroid hormones, and albuminuria in T2DM patients. The findings demonstrated that CGM-based metrics, including mean of glucose, SD of glucose, CV of glucose, and MAGE, were associated with higher odds of albuminuria. These relationships persisted in multivariate models adjusted for biochemical factors, medication-related information, disease-related variables, and demographic characteristics.

Higher variation in these CGM-derived metrics was associated with albuminuria in the present study. Previous work has established the clinical value of CGM-derived metrics for characterizing glycemic exposure [3,10-14], while studies specifically examining renal outcomes have reported associations between glycemic variability and albuminuria [4,11]. Regarding TIR, the present study found no significant association with albuminuria or macroalbuminuria. Luo et al. similarly reported a non-significant association of TIR with albuminuria [4].

HbA1c remains an established marker of long-term glycemic exposure, although it does not fully capture short-term glycemic variability. In the present analysis, higher HbA1c was associated with albuminuria. Long-term glycemic exposure has also been linked to microvascular complications in major longitudinal diabetes studies [15,16]. A past study presented a comprehensive discussion about the complications, prevention, and treatment of diabetes. The author stated that a 5.5-9.5% increase in HbA1c causes an increment of approximately 10-fold higher risk of microvascular complications and nearly twofold additional risk of macrovascular disease [2]. Another study conducted in 2017 addressed susceptible, initiatory, and progressive risk factors of kidney-specific complications in diabetic patients. The authors specified target limits of HbA1c according to age, duration of diabetes, and presence of comorbidities to prevent microvascular diseases in diabetic patients [1].

Hypomagnesemia is considered the most frequent disorder associated with diabetes mellitus due to various causes. Several studies have identified that magnesium deficiency is related to increased probability of developing T2DM and progression of DN. Experiments even evidenced that higher magnesium intake is associated with lower fasting measures of insulin for both diabetic and non-diabetic populations [17-20]. The univariate analysis of the present study evidenced that hypomagnesemia is significantly associated with higher odds of albuminuria. Serum magnesium was inversely associated with albuminuria in Models I-III, but the association was attenuated and no longer statistically significant after demographic adjustment in Model IV (p=0.060).

Integrated with CGM-based metrics and magnesium level, the present analysis also investigated the relationship of thyroid hormones with albuminuria. FT3 showed a significant association with lower odds of albuminuria in univariate and multivariate analyses even after adjustment of biochemical, medication-related, disease-related, and demographic factors. The literature reported a higher prevalence rate of abnormal thyroid hormones in diabetic patients compared to non-diabetic individuals [21]. Additionally, thyroid hormones are reported as a significant determinant of DKD [22]. Consistent with the present study, several studies conducted in different countries with diverse populations evidenced that lower FT3 levels are associated with the progression of DKD [23-25]. The present analysis found no significant association of FT4 and TSH with the odds of albuminuria or macroalbuminuria. Several studies investigated the relationship between FT4 and TSH, with the prevalence of albuminuria showing a non-significant effect of these thyroid hormones [4,26]. However, some studies showed a significant association of TSH with a higher risk of albuminuria regardless of gender [27]. Consistent with past studies, the present analysis observed no significant association of HbA1c, mean of glucose, SD of glucose, CV of glucose, MAGE, and serum magnesium with macroalbuminuria [4]. 

Exploratory mediation analysis identified statistically significant indirect associations through FT3 for HbA1c and glucose SD. FT3 statistically accounted for 6.9% of the HbA1c-albuminuria association and 21.4% of the glucose SD-albuminuria association. No significant indirect associations through FT4 or TSH were observed. Given the cross-sectional design, these findings should be interpreted as exploratory statistical mediation rather than evidence of a temporal or causal pathway.

In 2026, Luo et al. reported non-significant statistical mediation through FT3, FT4, and TSH in the relationships between several glycemic indices and albuminuria [4]. In contrast, the present study identified modest statistically significant indirect associations through FT3 for HbA1c and glucose SD, whereas FT4 and TSH showed no significant indirect associations. Evidence evaluating thyroid hormones within these glycemic-albuminuria relationships remains limited [4,28-30].

Some limitations should be acknowledged about this research study. First, the cross-sectional design does not allow determination of temporal relationships between albuminuria, thyroid function, and glycemic control. The mediation findings should therefore be interpreted as exploratory statistical associations rather than evidence of a temporal or causal pathway. Second, the generalizability of the findings may be limited by the single-center design. Third, residual confounding from unmeasured variables, such as medication dose or duration, lifestyle characteristics, and dietary factors, cannot be excluded despite adjustment for measured covariates. UACR was used to classify albuminuria status at the study assessment and was not intended to establish longitudinal persistence of albuminuria. The macroalbuminuria subgroup was relatively small (n=24), and findings from this subgroup should therefore be considered exploratory. Prospective multicenter studies including larger and more diverse populations are required to clarify the temporal relationships and clinical significance of these findings.

## Conclusions

Greater glycemic variability and lower FT3 levels were independently associated with albuminuria in patients with T2DM, while hypomagnesemia was associated with albuminuria in univariable analysis. Exploratory mediation analysis suggested that FT3 statistically accounted for a modest proportion of the associations of HbA1c and glucose SD with albuminuria. These findings indicate that CGM-derived variability measures, alongside magnesium status and thyroid function, may provide complementary information in the assessment of DKD. Prospective multicenter studies are required to clarify the temporal relationships and clinical significance of these findings.

## Figures and Tables

**Figure 1:**
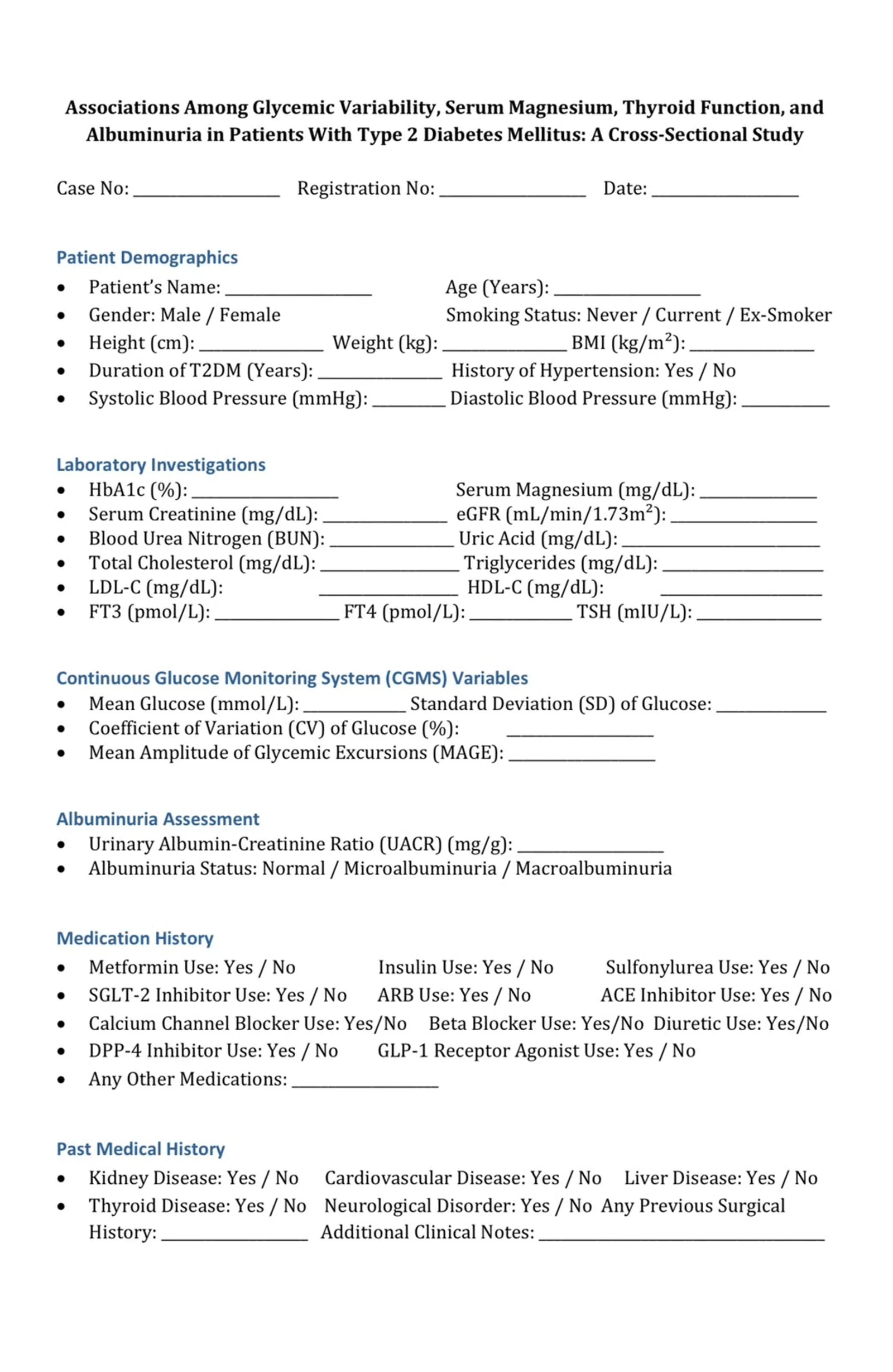
Research proforma

## References

[1] Alicic RZ, Rooney MT, Tuttle KR (2017). Diabetic kidney disease: challenges, progress, and possibilities. Clin J Am Soc Nephrol.

[2] Samsu N (2021). Diabetic nephropathy: challenges in pathogenesis, diagnosis, and treatment. Biomed Res Int.

[3] Battelino T, Danne T, Bergenstal RM (2019). Clinical targets for continuous glucose monitoring data interpretation: recommendations from the International Consensus on Time in Range. Diabetes Care.

[4] Luo N, Feng SY, Chen H (2026). Correlations among glycemia and glucose variability, thyroid hormones, and albuminuria in patients with type 2 diabetes mellitus. Kaohsiung J Med Sci.

[5] Pham PC, Pham PM, Pham SV, Miller JM, Pham PT (2007). Hypomagnesemia in patients with type 2 diabetes. Clin J Am Soc Nephrol.

[6] Dasgupta A, Sarma D, Saikia UK (2012). Hypomagnesemia in type 2 diabetes mellitus. Indian J Endocrinol Metab.

[7] Barbagallo M, Dominguez LJ (2015). Magnesium and type 2 diabetes. World J Diabetes.

[8] Chen HS, Wu TE, Jap TS, Lu RA, Wang ML, Chen RL, Lin HD (2007). Subclinical hypothyroidism is a risk factor for nephropathy and cardiovascular diseases in type 2 diabetic patients. Diabet Med.

[9] Sana MA, Chaudhry M, Malik A, Iqbal N, Zakiuddin A, Abdullah M (2020). Prevalence of microalbuminuria in type 2 diabetes mellitus. Cureus.

[10] Riddlesworth TD, Beck RW, Gal RL, Connor CG, Bergenstal RM, Lee S, Willi SM (2018). Optimal sampling duration for continuous glucose monitoring to determine long-term glycemic control. Diabetes Technol Ther.

[11] Wakasugi S, Mita T, Katakami N (2021). Associations between continuous glucose monitoring-derived metrics and diabetic retinopathy and albuminuria in patients with type 2 diabetes. BMJ Open Diabetes Res Care.

[12] Beck RW, Riddlesworth TD, Ruedy K (2017). Continuous glucose monitoring versus usual care in patients with type 2 diabetes receiving multiple daily insulin injections: a randomized trial. Ann Intern Med.

[13] Fonseca VA, Grunberger G, Anhalt H (2016). Continuous glucose monitoring: a consensus conference of the American Association of Clinical Endocrinologists and American College of Endocrinology. Endocr Pract.

[14] Danne T, Nimri R, Battelino T (2017). International consensus on use of continuous glucose monitoring. Diabetes Care.

[15] Stratton IM, Adler AI, Neil HA (2000). Association of glycaemia with macrovascular and microvascular complications of type 2 diabetes (UKPDS 35): prospective observational study. BMJ.

[16] (1995). The relationship of glycemic exposure (HbA1c) to the risk of development and progression of retinopathy in the diabetes control and complications trial. Diabetes.

[17] Yokota K (2005). Diabetes mellitus and magnesium [Article in Japanese]. Clin Calcium.

[18] Longstreet DA, Heath DL, Vink R (2005). A potential link between magnesium intake and diabetes in Indigenous Australians. Med J Aust.

[19] Tin A, Grams ME, Maruthur NM (2015). Results from the Atherosclerosis Risk in Communities study suggest that low serum magnesium is associated with incident kidney disease. Kidney Int.

[20] Fung TT, Manson JE, Solomon CG, Liu S, Willett WC, Hu FB (2003). The association between magnesium intake and fasting insulin concentration in healthy middle-aged women. J Am Coll Nutr.

[21] Papazafiropoulou A, Sotiropoulos A, Kokolaki A, Kardara M, Stamataki P, Pappas S (2010). Prevalence of thyroid dysfunction among Greek type 2 diabetic patients attending an outpatient clinic. J Clin Med Res.

[22] Iglesias P, Bajo MA, Selgas R, Díez JJ (2017). Thyroid dysfunction and kidney disease: an update. Rev Endocr Metab Disord.

[23] Wu J, Li X, Tao Y, Wang Y, Peng Y (2015). Free triiodothyronine levels are associated with diabetic nephropathy in euthyroid patients with type 2 diabetes. Int J Endocrinol.

[24] Sun MT, Hsiao FC, Su SC, Pei D, Hung YJ (2012). Thyrotropin as an independent factor of renal function and chronic kidney disease in normoglycemic euthyroid adults. Endocr Res.

[25] Chen Y, Zhang W, Wang N, Wang Y, Wang C, Wan H, Lu Y (2020). Thyroid parameters and kidney disorder in type 2 diabetes: results from the METAL study. J Diabetes Res.

[26] Yang Z, Duan P, Li W (2022). The correlation between thyroid hormone levels and the kidney disease progression risk in patients with type 2 diabetes. Diabetes Metab Syndr Obes.

[27] Gao J, Liu J (2024). Correlation of serum thyrotropin and thyroid hormone levels with diabetic kidney disease: a cross-sectional study. BMC Endocr Disord.

[28] Biondi B, Kahaly GJ, Robertson RP (2019). Thyroid dysfunction and diabetes mellitus: two closely associated disorders. Endocr Rev.

[29] Liu J, Jiang W, Liang J (2026). Association between thyroid hormones and diabetic kidney disease in euthyroid type 2 diabetes mellitus patients. Front Endocrinol (Lausanne).

[30] Zhang Y, Wang Y, Kang Q (2025). The role of advanced glycation end products between thyroid function and diabetic nephropathy and metabolic disorders. Sci Rep.

